# Potent and Persistent Antibody Response in COVID-19 Recovered Patients

**DOI:** 10.3389/fimmu.2021.659041

**Published:** 2021-05-28

**Authors:** Xiaodong Tian, Ling Liu, Wenguo Jiang, He Zhang, Wenjun Liu, Jing Li

**Affiliations:** ^1^ CAS Key Laboratory of Pathogenic Microbiology and Immunology, Institute of Microbiology, Chinese Academy of Sciences, Beijing, China; ^2^ School of Life Sciences, University of Science and Technology of China, Hefei, China; ^3^ Institutes of Physical Science and Information Technology, Anhui University, Hefei, China; ^4^ Jining Center for Disease Control and Prevention, Shandong, China; ^5^ Savaid Medical School, University of Chinese Academy of Sciences, Beijing, China; ^6^ Institute of Microbiology, Center for Biosafety Mega-Science, Chinese Academy of Sciences, Beijing, China

**Keywords:** COVID-19, SARS-CoV-2, antibody response, recovered patients, persistent, potent

## Abstract

SARS-CoV-2 has caused a global pandemic with millions infected and numerous fatalities. Virus-specific antibodies can be detected in infected patients approximately two weeks after symptom onset. In this study, we set up ELISA technology coating with purified SARS-CoV-2 S and N proteins to study the antibody response of 484 serum samples. We established a surrogate viral inhibition assay using SARS-CoV-2 S protein pseudovirus system to determine the neutralization potency of collected serum samples. Here, we report robust antibody responses to SARS-CoV-2 in 484 recovered patients varying from 154 to 193 days, with 92% of recovered patients displaying a positive virus-specific spike glycoprotein IgG (s-IgG) response, while the ratio of positive spike glycoprotein IgM (s-IgM) reached 63%. Furthermore, moderate to potent neutralization activities were also observed in 62% of patients, correlating significantly with s-IgG response. This study strongly supports the long-term presence of antibodies in recovered patients against SARS-CoV-2, although all serum samples were collected from individuals with mild or moderate symptoms.

The outbreak of severe acute respiratory syndrome coronavirus 2 (SARS-CoV-2) in late 2019 has caused a global pandemic, posing serious threats to global public health, social stability, and economic development. It is critical to monitor immune response parameters in both recently infected and convalescent patients to understand disease progression, make effective prognostic judgments, and provide targeted immunotherapy and information for vaccine development. In this study, hundreds of blood samples from convalescent patients were studied over a five to six-month after SARS-CoV-2 infection. Serum titers and neutralizing activities were also investigated to better understand the immune response of patients recovering from SARS-CoV-2 infection.

SARS-CoV-2 pandemic has caused millions of people infection and numerous fatalities. Disease severity is highly correlated with viral infection characteristics, antibody response, and population diversity (1). The previous study demonstrated patients with severe disease induced higher antibody levels than those with non-severe disease; however, a significant difference in IgG antibody levels was observed only in the early stages of the disease between the severe and non-severe cases (2). In addition, a serological survey of 175 mild cases demonstrated that neutralizing antibodies were produced 10-15 days after the onset of SARS-CoV-2 infection and that middle-aged and elderly patients produced much higher neutralizing antibody titers than young patients. However, 30% of patients exhibited very low antibody titers; among these, the antibody titers of ten convalescent patients failed to reach the detection limit, highlighting an important limitation to mapping the epidemiology of SARS-CoV-2 infection by antibody detection (3, 4). As the SARS-CoV-2 pandemic emerged, quite a few asymptomatic cases of SARS-CoV-2 infection were observed, rendering it critical to investigate the immunological characteristics of such asymptomatic cases (5). Previous study shown that IgG antibody levels of the asymptomatic group were significantly lower than those of the symptomatic group in both the acute infection stage and the convalescent stage. The antibody levels in about 90% of the cases had decreased by 70% or more two months after discharge, suggesting that serological testing should be carried out as soon as possible. Furthermore, decreased neutralizing antibody levels were also observed in about 80% and 69% of asymptomatic and symptomatic cases, respectively, with an average reduction of about 8% and 11%, respectively (6). However, the recently published research demonstrated that SARS-CoV-2 infected hospitalized patients displayed durable and stable antibodies response (7–9). SARS-CoV-2 infected asymptomatic patients shown the similar trend, although the neutralizing antibody titers were lower compared with confirmed cases and symptomatic individuals (10).

Due to their specificity and high affinity, neutralizing antibodies play a role in protecting host cells from invasion by neutralizing or inhibiting the biological activity of pathogens, suggesting that neutralizing antibodies may be used as both prophylactic and therapeutic drugs in high-exposure situations (11, 12). At present, multiple monoclonal antibodies-screened and identified by high-throughput single-cell sequencing and fluorescence-activated cell sorting-have proven to be effective against the SARS-CoV-2 spike protein-receptor-binding domain (RBD), revealing that anti-SARS-CoV-2 spike protein-RBD monoclonal antibodies may serve as potential therapeutic candidates for SARS-CoV-2 infection (13–16). Moreover, a recently published study found that numerous antibodies in patients also played a neutralizing role without binding to the RBD, indicating that the use of highly active antiretroviral therapy-commonly known as “cocktail therapy”-may have greater therapeutic potential in combination with multiple antibodies against different antigenic epitopes (17–19).

Monitoring the immune response in infected and convalescent patients is critical to analyzing the pathogenic mechanism of SARS-CoV-2 and guiding clinical diagnosis and treatment (20, 21). In this study, hundreds of serum samples were collected from convalescent patients who were recovered from SARS-CoV-2 infection over a five to six-month after. Serum titers and neutralizing activities were investigated to understand the immune response of patients recovering from SARS-CoV-2 infection. A total of 484 patients recovered with COVID-19 were enrolled in this study. The characteristics of these patients are summarized in **Supplemental Table 1**. All of these cases were in the hospital and correctional facility of Shandong province recovered from 154 to 193 days after diagnosis of illness. To study the antibody response to SARS-CoV-2, the IgG and IgM responses against S glycoprotein and N protein were measured by enzyme-linked immunosorbent assay (ELISA). Initially, the OD_450_ at a 1:400 serum dilution was measured for 484 samples. We used the 30 healthy human serum collected before the outbreak of COVID-19 serves as negative control. The characteristics of negative controls are summarized in **Supplemental Table 2**. The mean OD value of the negative control samples plus 2.1 standard deviations (SDs) was set up as cutoff value. The cutoff values of IgG and IgM against S protein at 0.140 and 0.154, respectively. For N protein, the cutoff value of IgG and IgM was 0.156 and 0.153. All 484 individuals generated detectable antibody responses again S and N antigens over the follow-up period. The proportion of recovered patients with positive virus-specific s-IgG reached approximately 93% (451/484), while the proportion with positive s-IgM reached approximately 70% (338/484). The frequency of recovered patients with IgG and IgM responses to N protein was lower, with only 76% (367/484) and 48% (230/484) seropositive, respectively (**Figures 1A–D**).

**Figure 1:**
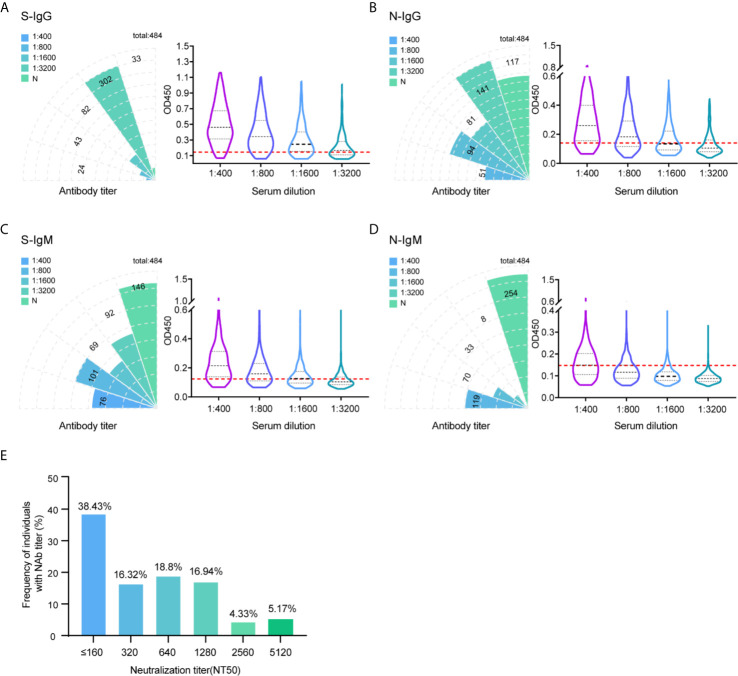
Profile of IgG and IgM and neutralization antibody response in 484 recovered patients infected with SARS-CoV-2. The absolute positive numbers of individuals with S-IgG **(A)**, N-IgG **(B)**, S-IgM **(C)** and N-IgM **(D)** antibody titers of non-detected (N), 1:400 (low), 1:800 (moderate), 1:1600 (high), and 1:3200 (very high). Testing of each sample was performed using ELISA assay. The corresponding OD450 values at different serum dilution were shown in violin plot. 30 healthy human serum collected before the outbreak of COVID-19 serves as negative control. Red dashed line denoted the cut-off value. A serum sample is considered positive when the OD is above the cut-off value. **(E)** Neutralization activity of 484 patients serums in different dilution were displayed. The x-axis indicates the values that the serum titers at which 50% neutralization (NT50) was recorded. The y-axis values represent the frequency of individuals with neutralization activity.

To further differentiate the antibody response against the SARS-CoV-2 virus, in addition to the results measured at 1:400 titer, ELISA was also performed in discrete ether titers of 1:800, 1:1600, and 1:3200. The 1:400 titer was categorized as low, 1:800 as moderate, 1:1600 as high, and 1:3200 as very high titers. Of the 451 IgG positive samples against S protein, 24 (5.3%) had a titer of 1:400, 43 (9.5%) of 1:800, 82 (18.2%) of 1:1600, and 302 (67%) of 1:3200 (**Figure 1A**). Thus, the majority of positive individuals had high to very high titers of anti-spike antibodies.

Determining the neutralizing effects of SARS-CoV-2 spike antibodies is critical to understand the possible protective effects of the immune response. Therefore, we measured SARS-CoV-2 neutralization potency using a surrogate viral inhibition assay that utilized lentivirus-based virus particles, pseudo-typed with the S protein of SASR-CoV-2 and 293T cells stably expressing hACE-2 receptor. All 484 individuals generated detectable neutralizing antibody responses. Of the 484 samples, 38% (NT50 value < 320) had low, 35% (NT50 value 320-640) had medium, 17% (NT50 value 1280) had high, and 9.5% (NT50 value 2560-5120) had potent neutralizing titers (**Figure 1E**). When considering candidates for plasma therapy, titers of 1:320 or higher were initially deemed eligible (22); 62% of serum samples displayed moderate to potent neutralization activities, indicating detectable antibody responses up to 193 days during the follow-up period.

To investigate whether serology testing correlated with sera neutralization activities, the Spearman’s correlation was calculated between serum antibodies against SARS-CoV-2 S/N proteins and neutralization activity (**Figure 2**). All sera antibodies displayed positive correlation with neutralization antibody. The highest Spearman’s correlation of 0.597 was observed between S-IgG and neutralization antibody (*p* < 0.001). The significant positive correlations between S/N IgG and S/N IgM were also noted. The correlations between S-IgG and N-IgG were highest with Spearman’s correlation coefficients of up to 0.593 (*p* < 0.001).

**Figure 2:**
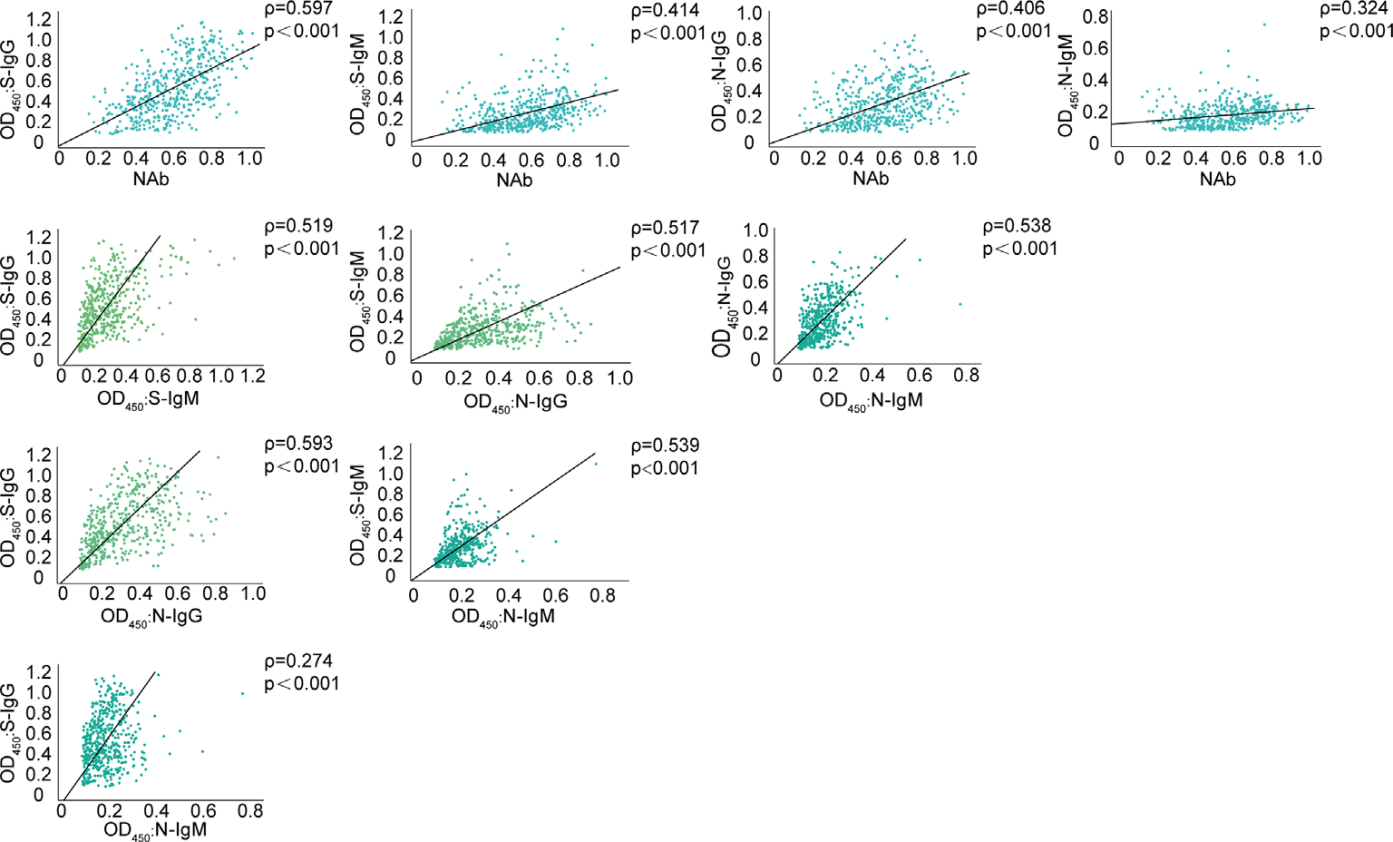
Correlation between serum antibody against SARS-CoV-2 S/N proteins and neutralization activity or serum antibodies. The correlation between serum IgG and IgM antibodies against S/N proteins and neutralization activity or serum antibodies were analyzed using spearman analysis. 484 serum samples at 1: 400 dilution from recovered patients were detected using ELISA assay. The neutralization antibody titer was also measure at 1:320 dilution. Spearman correlation coefficients are depicted in plots.

To compare the antibody response of mild and moderate COVID-19 patients, all 484 patients were separated into mild and moderate group according to the criteria of mild and moderate COVID-19, the mild patients usually presented mild non-to-mild clinical symptoms; the moderate COVID-19 patients had fever and respiratory symptoms. 340 patients were included in mild group and 144 patients were included in moderate group. We compared the proportion of recovered patients with positive virus-specific s-IgG/s-IgM/N-IgG/N-IgM between the two groups, no big difference was displayed. The similar proportion of neutralization antibody response were also displayed between these two groups, indicating even mild-moderate COVID-19 patients induce substantial antibody response. To assess whether the antibody response can predict the clinical mild to moderate symptoms, the Spearman’s correlation analyses were also performed to compare serum antibodies against SARS-CoV-2 S/N proteins and neutralization activity in these two groups (**Figure 3**). Unfortunately, no significant difference was detected between two groups.

**Figure 3:**
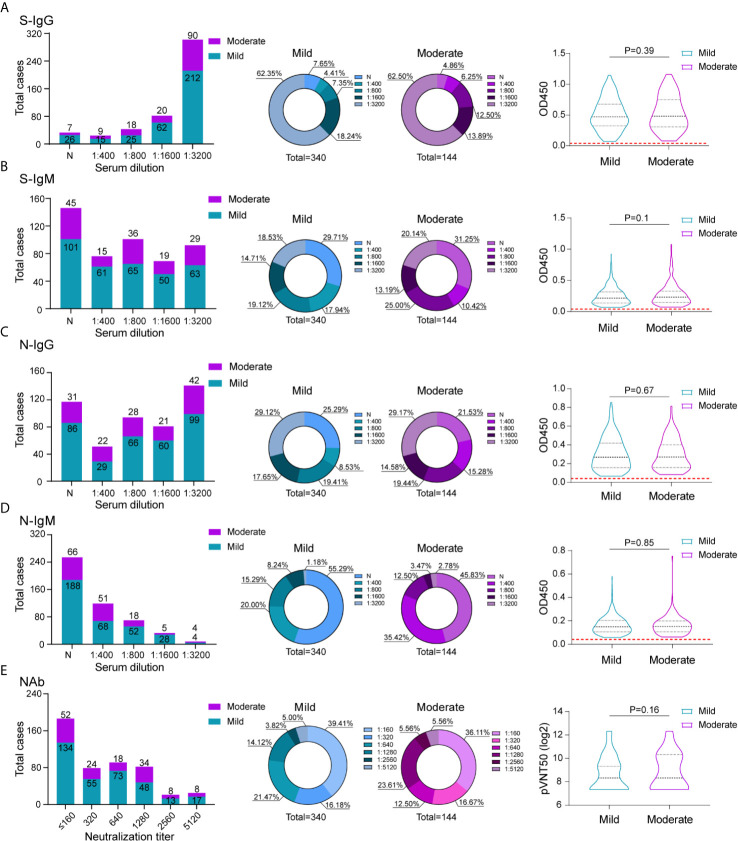
Comparison of antibody response between mild and moderate COVID-19 patients. The absolute and proportion positive numbers of mild and moderate patients with S-IgG **(A)**, N-IgG **(B)**, S-IgM **(C)** and N-IgM **(D)** antibody titers of non-detected (N), 1:400 (low), 1:800 (moderate), 1:1600 (high), and 1:3200 (very high). Testing of each sample was performed using ELISA assay. The corresponding OD450 values at different serum dilution were shown in violin plot. Red dashed line denoted the cut-off value. **(E)** Neutralization activity of serums in different dilution between mild and moderate patients were displayed. The values are the serum titers at which 50% neutralization (NT50) was recorded. The correlation of serum S-IgG **(A)**, N-IgG **(B)**, S-IgM **(C)**N-IgM **(D)** and neutralization antibody activity **(E)** between mild and moderate patients were analyzed using spearman analysis.

It remains a mystery whether SARS-CoV-2 infection in humans protects from reinfection and-if so-for how long; it is also unknown how long vaccine-induced antibodies might last (23–27). The results of our study indicated that individuals who have recovered from mild-to-moderate symptoms generate robust antibody responses to the S protein, which is highly correlated with neutralization of the SARS-CoV-2 virus. Furthermore, we identified high antibody titers-especially S-IgG, which can be detected up to five to six months. Interestingly, we did not observe a decrease beyond the six-month time point, indicating a long-term presence of antibodies against SARS-CoV-2. There are several limitations in our study. Given that all serum samples were collected from individuals with moderate or mild symptoms, it is difficult to determine the correlation between antibody response and clinical severe disease course. Although we assessed the relationship of antibody response with mild to moderate COVID-19 patients. No statistically correlation were shown in our study. A single time-point sample-collection protocol also limited our understanding of the kinetic antibody response during SARS-CoV-2 infection. Repeat sampling of the same patients over extended periods of time should be performed in future studies to better understand long-term antibody responses against SARS-CoV-2.

## Methods

### Ethics Statement

All the experiments were carried out according to the procedures approved by the Institute of Microbiology, Chinese Academy of Sciences and complied with all relevant ethical regulations regarding animal research.

### Cell Lines

The human embryonic 293T cell line and human cells adapted in suspension (293-F cells) were stored in our laboratory. The 293T cells stably expressing hACE2 (293T/hACE2) were kindly provided by Dr. Zhendong Zhao (Institute of Pathogen Biology, Chinese Academy of Medical Sciences & Peking Union Medical College) (28). All cells were cultured in DMEM medium supplemented with 10% FBS (Cat# 26140079, Thermo Fisher Scientific) in 5% CO2 at 37°C.

### Human Samples and Serum Collection

The human samples were obtained according to procedures approved by the Chinese Academy of Sciences, and complied with all relevant ethical regulations regarding human research. The blood was taken from the patient convalescing from COVID-19 after they had signed the informed consent form. A total of 484 serum samples were collected from prior SARS-CoV-2 nucleic acid-positive and recovered patients in hospital and correctional facility of Shandong province, China.

### Production and Titration of SARS-CoV-2 Pseudovirions

To produce pseudovirions, pLenti-GFP, psPAX2, and plasmids encoding SARS-CoV-2 S were co-transfected into 293F cells using polyetherimide (PEI) (Cat#40816ES03, Shanghai YEASEN Biotechnology). The cells were maintained by adding fresh medium every 48 hours. The supernatants were harvested at 5 days post transfection, passed through 0.45 μm filters and centrifuged at 2000 × *g* for 10 minutes to remove cell debris. The supernatant containing pseudovirions were stored at –80°C for further use. For titration of the pseudovirus, 293T/hACE2 cells were pre-plated in a 96-well plate, then the pseudovirus was diluted 5 times and each dilution contain 4 parallel control. The last column serves as the cell control without the addition of pseudovirus. After 40h incubation, 100μl of luciferase reporter substrate (Cat# RG051M, Sino Biological) was added to detect luminescence using a microplate luminometer (GloMax 96, Promega). The 50% tissue culture infection dose (TCID_50_) was calculated using Reed-Muench method.

## ELISA

ELISA was performed to evaluate the binding of antibodies to SARS-CoV-2 S/N proteins (S protein: Cat#40589-V08B1; N protein: Cat#40588-V08B, Beijing Sino Biological) by coating high-binding 96-well plates with 100-μL per well of 1 μg/mL protein solution in PBS overnight at 4°C. The plates were washed three times with PBST solution and incubated with blocking buffer containing 2% BSA and 3% sucrose at 4°C overnight. The prepared plates were vacuumized and stored at 4°C for further use. For antibody detection, the serum samples were incubated at 56°C for 30 minutes to inactivate the complements. The serum samples were prepared at a 1:100 dilution and five additional two-fold serial dilutions. The serially diluted samples were added to the ELISA plate at 50 μL per well. The plates were incubated at 37°C for 2 hours. The plates were washed six times with washing buffer, followed by incubation with 50 μL diluent of secondary antibodies; goat anti-human IgG-HRP (Cat#P03S121S, Beijing Gene-protein Link) and goat anti-human IgM-HRP (Cat#P03S108S, Beijing Gene-protein Link) (1:5000) were added at 37°C for one hour. After six washes, 100 μL TMB (Cat#PA107-01, Beijing Tiangen) substrate solution was added to the plates at 37°C for 15 minutes, then mixed immediately with 100 μL stop solution. Absorbance was measured at 450 nm. To set up ELISA cut-off values, 30 healthy human serum collected before the outbreak of COVID-19 serves as negative control. The samples are processed as follows: OD450 <0.1 was initially confirmed as quality control, then the mean OD450 value plus 2.1 times the standard deviation was obtained as the cut-off value of the corresponding antibody (29, 30): the cut-off value for S-IgG was 0.140, for S-IgM was 0.154, for N-IgG was 0.156, for N-IgM was 0.153. The serum sample is considered positive when the OD is above the cut-off value.

### SARS-CoV-2 Pseudovirus Neutralization Assay

Human 293T cells stably expressing hACE2 were inoculated in a 96-well plate 24 hours before the experiment. The serum samples were heat-inactivated at 56°C for 30 minutes prior to use. Beginning with a 1:10 dilution, twofold serial dilutions of each sample were prepared in a 96-well plate. Equal volumes of SARS-CoV-2 pseudovirus particles were mixed with each diluted serum sample and incubated at 37°C for 1 hour. The virus-serum mixture was then added to the 293T/hACE2 cells. After incubation for 6 hours, the mixture was removed and changed to fresh medium. Forty hours later, firefly luciferase activity in the cells was detected by chemiluminescence and the luciferase activity was quantified to measure the transduction efficiency. To calculate neutralization efficiency, the same dose of pseudovirus (without antibody) serves as positive control. The positive value was determined as ten-fold relative luminescence unit (RLU) values higher than the cell only background. The half-maximal neutralization titer (NT50) value was calculated by the luciferase activity.

## Statistical Analysis

Data analysis was performed using GraphPad and SPSS. The data were annotated, the correlation between different antibodies was analyzed using *Spearman’s rank test*, and the significance of the correlation coefficient was verified.

## Data Availability Statement

The original contributions presented in the study are included in the article/**Supplementary Material**. Further inquiries can be directed to the corresponding authors.

## Ethics Statement

The studies involving human participants were reviewed and approved by the Institute of Microbiology, Chinese Academy of Sciences. The patients/participants provided their written informed consent to participate in this study.

## Author Contributions

JL conceived and designed the experiments. XT performed the ELISA tests, neutralization assay, and statistics analyses. LL and HZ performed other experimental data analyses. WJ performed the serum collection. JL and XT wrote the manuscript and completed its revision. WL suggested many of the experiments in this study. All authors contributed to the article and approved the submitted version.

## Funding

This work has been supported by the grants from the Strategic Priority Research Program of the Chinese Academy of Sciences (XDB29010000), the National Natural Science Foundation of China (31970153), the National Key R&D Program of China (2016YFD0500206), and Youth Innovation Promotion Association of CAS (2019091).

## Conflict of Interest

The authors declare that the research was conducted in the absence of any commercial or financial relationships that could be construed as a potential conflict of interest.
