# Supplementary material for: Potent and Persistent Antibody Response in COVID-19 Recovered Patients
Source: Front Immunol. 2021 May 28;12:659041. doi: 10.3389/fimmu.2021.659041 (PMC8193946; doi:10.3389/fimmu.2021.659041)
Supplement: Supplementary file 1 [file Table_1.doc]

**Supplemental Table 1. The characteristics of 484 patients recovered with COVID-19 were enrolled in this study**

| **No.** | **Sex** | **qPCR** | **S-IgM (OD450) (1:400)** | **S-IgG (OD450) (1:400)** | **N-IgM (OD450) (1:400)** | **N-IgG (OD450) (1:400)** | **Neutralization activities (NT50)** | **Disease phenotype** | **Time from diagnosis (Day)** |
| --- | --- | --- | --- | --- | --- | --- | --- | --- | --- |
| 248 | M | + | 1.076 | 0.979 | 0.753 | 0.431 | 1/640 | Mild symptoms | 193 |
| 301 | F | + | 0.304 | 0.895 | 0.304 | 0.509 | 1/320 | Mild symptoms | 193 |
| 370 | M | + | 0.154 | 0.288 | 0.082 | 0.135 | 1/160 | Mild symptoms | 193 |
| 383 | F | + | 0.241 | 0.534 | 0.088 | 0.25 | 1/160 | Moderate symptoms | 193 |
| 331 | F | + | 0.14 | 0.554 | 0.094 | 0.11 | 1/160 | Mild symptoms | 193 |
| 392 | F | + | 0.112 | 0.427 | 0.095 | 0.333 | 1/160 | Moderate symptoms | 193 |
| 309 | F | + | 0.232 | 0.748 | 0.189 | 0.492 | 1/160 | Moderate symptoms | 193 |
| 177 | M | + | 0.102 | 0.187 | 0.232 | 0.26 | 1/160 | Moderate symptoms | 193 |
| 318 | M | + | 0.282 | 0.632 | 0.143 | 0.427 | 1/1280 | Moderate symptoms | 193 |
| 269 | M | + | 0.313 | 0.813 | 0.246 | 0.615 | 1/1280 | Mild symptoms | 193 |
| 406 | F | + | 0.167 | 0.125 | 0.114 | 0.117 | 1/640 | Mild symptoms | 192 |
| 302 | F | + | 0.774 | 0.939 | 0.187 | 0.35 | 1/640 | Mild symptoms | 192 |
| 312 | M | + | 0.412 | 0.856 | 0.205 | 0.523 | 1/640 | Mild symptoms | 192 |
| 171 | M | + | 0.145 | 0.697 | 0.246 | 0.423 | 1/640 | Mild symptoms | 192 |
| 420 | M | + | 0.152 | 0.19 | 0.09 | 0.183 | 1/320 | Moderate symptoms | 192 |
| 483 | M | + | 0.17 | 0.643 | 0.115 | 0.129 | 1/320 | Mild symptoms | 192 |
| 137 | M | + | 0.267 | 0.441 | 0.183 | 0.21 | 1/320 | Moderate symptoms | 192 |
| 167 | M | + | 0.086 | 0.151 | 0.221 | 0.177 | 1/320 | Mild symptoms | 192 |
| 201 | M | + | 0.17 | 0.782 | 0.136 | 0.481 | 1/2560 | Mild symptoms | 192 |
| 245 | M | + | 0.733 | 0.989 | 0.254 | 0.39 | 1/2560 | Mild symptoms | 192 |
| 375 | M | + | 0.118 | 0.419 | 0.076 | 0.268 | 1/160 | Mild symptoms | 192 |
| 338 | F | + | 0.154 | 0.594 | 0.081 | 0.119 | 1/160 | Mild symptoms | 192 |
| 442 | M | + | 0.154 | 0.489 | 0.096 | 0.292 | 1/160 | Moderate symptoms | 192 |
| 146 | M | + | 0.235 | 0.47 | 0.162 | 0.184 | 1/160 | Mild symptoms | 192 |
| 308 | M | + | 0.241 | 0.774 | 0.224 | 0.575 | 1/160 | Mild symptoms | 192 |
| 282 | M | + | 0.374 | 0.849 | 0.149 | 0.374 | 1/1280 | Moderate symptoms | 192 |
| 256 | M | + | 0.276 | 0.962 | 0.269 | 0.433 | 1/1280 | Moderate symptoms | 192 |
| 449 | M | + | 0.146 | 0.618 | 0.065 | 0.299 | 1/640 | Mild symptoms | 191 |
| 451 | M | + | 0.188 | 0.386 | 0.076 | 0.162 | 1/640 | Mild symptoms | 191 |
| 432 | F | + | 0.158 | 0.448 | 0.079 | 0.286 | 1/640 | Mild symptoms | 191 |
| 49 | F | + | 0.424 | 0.675 | 0.226 | 0.197 | 1/640 | Mild symptoms | 191 |
| 229 | M | + | 0.266 | 0.575 | 0.249 | 0.527 | 1/640 | Mild symptoms | 191 |
| 341 | M | + | 0.307 | 0.601 | 0.108 | 0.157 | 1/320 | Mild symptoms | 191 |
| 295 | M | + | 0.663 | 1.101 | 0.152 | 0.447 | 1/320 | Mild symptoms | 191 |
| 369 | M | + | 0.111 | 0.365 | 0.081 | 0.148 | 1/160 | Mild symptoms | 191 |
| 393 | F | + | 0.158 | 0.28 | 0.094 | 0.139 | 1/160 | Mild symptoms | 191 |
| 150 | M | + | 0.18 | 0.346 | 0.149 | 0.146 | 1/160 | Mild symptoms | 191 |
| 334 | F | + | 0.425 | 0.878 | 0.106 | 0.372 | 1/1280 | Moderate symptoms | 191 |
| 124 | M | + | 0.358 | 0.361 | 0.214 | 0.223 | 1/640 | Mild symptoms | 190 |
| 424 | M | + | 0.13 | 0.378 | 0.099 | 0.183 | 1/320 | Moderate symptoms | 190 |
| 419 | F | + | 0.255 | 0.474 | 0.199 | 0.339 | 1/320 | Moderate symptoms | 190 |
| 47 | F | + | 0.191 | 0.382 | 0.124 | 0.217 | 1/2560 | Mild symptoms | 190 |
| 356 | M | + | 0.096 | 0.136 | 0.071 | 0.124 | 1/160 | Mild symptoms | 190 |
| 390 | F | + | 0.233 | 0.316 | 0.109 | 0.111 | 1/160 | Mild symptoms | 190 |
| 298 | M | + | 0.187 | 0.664 | 0.168 | 0.483 | 1/160 | Mild symptoms | 190 |
| 249 | M | + | 0.665 | 0.82 | 0.161 | 0.477 | 1/1280 | Mild symptoms | 190 |
| 123 | M | + | 0.31 | 0.424 | 0.197 | 0.219 | 1/1280 | Moderate symptoms | 190 |
| 57 | F | + | 0.44 | 0.8 | 0.21 | 0.248 | 1/1280 | Moderate symptoms | 190 |
| 106 | M | + | 0.185 | 0.599 | 0.135 | 0.317 | 1/640 | Mild symptoms | 189 |
| 114 | M | + | 0.202 | 0.902 | 0.136 | 0.231 | 1/640 | Mild symptoms | 189 |
| 243 | M | + | 0.278 | 0.791 | 0.196 | 0.499 | 1/5120 | Moderate symptoms | 189 |
| 288 | F | + | 0.386 | 0.675 | 0.177 | 0.374 | 1/320 | Mild symptoms | 189 |
| 235 | M | + | 0.371 | 0.613 | 0.482 | 0.658 | 1/320 | Mild symptoms | 189 |
| 455 | M | + | 0.084 | 0.121 | 0.061 | 0.08 | 1/160 | Mild symptoms | 189 |
| 358 | F | + | 0.156 | 0.572 | 0.089 | 0.157 | 1/160 | Mild symptoms | 189 |
| 382 | M | + | 0.151 | 0.508 | 0.117 | 0.475 | 1/160 | Mild symptoms | 189 |
| 408 | F | + | 0.336 | 0.428 | 0.152 | 0.28 | 1/160 | Mild symptoms | 189 |
| 156 | M | + | 0.089 | 0.204 | 0.156 | 0.154 | 1/160 | Mild symptoms | 189 |
| 65 | F | + | 0.301 | 0.424 | 0.139 | 0.174 | 1/1280 | Moderate symptoms | 189 |
| 149 | M | + | 0.352 | 0.349 | 0.229 | 0.362 | 1/1280 | Mild symptoms | 189 |
| 340 | M | + | 0.316 | 0.638 | 0.104 | 0.161 | 1/640 | Mild symptoms | 188 |
| 216 | M | + | 0.135 | 0.714 | 0.133 | 0.591 | 1/640 | Mild symptoms | 188 |
| 329 | M | + | 0.094 | 0.21 | 0.076 | 0.133 | 1/320 | Moderate symptoms | 188 |
| 189 | M | + | 0.146 | 0.344 | 0.132 | 0.216 | 1/320 | Mild symptoms | 188 |
| 465 | M | + | 0.082 | 0.09 | 0.062 | 0.114 | 1/160 | Mild symptoms | 188 |
| 107 | M | + | 0.233 | 0.317 | 0.118 | 0.392 | 1/160 | Mild symptoms | 188 |
| 175 | M | + | 0.091 | 0.157 | 0.159 | 0.074 | 1/160 | Mild symptoms | 188 |
| 94 | M | + | 0.288 | 0.223 | 0.22 | 0.332 | 1/160 | Mild symptoms | 188 |
| 179 | M | + | 0.132 | 0.205 | 0.257 | 0.394 | 1/160 | Mild symptoms | 188 |
| 320 | F | + | 0.46 | 0.88 | 0.124 | 0.272 | 1/1280 | Mild symptoms | 188 |
| 83 | F | + | 0.165 | 0.405 | 0.133 | 0.509 | 1/1280 | Moderate symptoms | 188 |
| 135 | M | + | 0.22 | 0.664 | 0.136 | 0.191 | 1/1280 | Mild symptoms | 188 |
| 353 | F | + | 0.182 | 0.463 | 0.102 | 0.12 | 1/640 | Moderate symptoms | 187 |
| 316 | F | + | 0.603 | 0.972 | 0.239 | 0.477 | 1/640 | Mild symptoms | 187 |
| 407 | M | + | 0.131 | 0.125 | 0.086 | 0.104 | 1/320 | Moderate symptoms | 187 |
| 349 | M | + | 0.454 | 0.587 | 0.104 | 0.407 | 1/320 | Mild symptoms | 187 |
| 155 | M | + | 0.175 | 0.582 | 0.149 | 0.122 | 1/320 | Mild symptoms | 187 |
| 472 | F | + | 0.073 | 0.099 | 0.065 | 0.077 | 1/160 | Mild symptoms | 187 |
| 426 | M | + | 0.114 | 0.235 | 0.08 | 0.136 | 1/160 | Mild symptoms | 187 |
| 151 | M | + | 0.183 | 0.267 | 0.141 | 0.136 | 1/160 | Moderate symptoms | 187 |
| 336 | M | + | 0.398 | 0.849 | 0.102 | 0.359 | 1/1280 | Mild symptoms | 187 |
| 87 | M | + | 0.209 | 0.416 | 0.149 | 0.23 | 1/1280 | Moderate symptoms | 187 |
| 136 | M | + | 0.312 | 0.476 | 0.211 | 0.188 | 1/1280 | Mild symptoms | 187 |
| 187 | M | + | 0.15 | 0.531 | 0.286 | 0.322 | 1/1280 | Mild symptoms | 187 |
| 467 | M | + | 0.084 | 0.362 | 0.066 | 0.135 | 1/640 | Mild symptoms | 186 |
| 350 | F | + | 0.139 | 0.362 | 0.086 | 0.149 | 1/640 | Mild symptoms | 186 |
| 281 | M | + | 0.194 | 0.686 | 0.128 | 0.377 | 1/640 | Mild symptoms | 186 |
| 199 | M | + | 0.162 | 0.66 | 0.139 | 0.461 | 1/640 | Mild symptoms | 186 |
| 48 | F | + | 0.342 | 0.637 | 0.185 | 0.199 | 1/640 | Mild symptoms | 186 |
| 469 | M | + | 0.085 | 0.105 | 0.067 | 0.084 | 1/160 | Mild symptoms | 186 |
| 365 | F | + | 0.163 | 0.66 | 0.099 | 0.477 | 1/160 | Mild symptoms | 186 |
| 386 | M | + | 0.227 | 0.615 | 0.122 | 0.132 | 1/160 | Mild symptoms | 186 |
| 50 | M | + | 0.253 | 0.345 | 0.17 | 0.211 | 1/160 | Mild symptoms | 186 |
| 440 | F | + | 0.157 | 0.694 | 0.228 | 0.337 | 1/160 | Moderate symptoms | 186 |
| 62 | M | + | 0.218 | 0.305 | 0.143 | 0.152 | 1/1280 | Moderate symptoms | 186 |
| 291 | F | + | 0.229 | 1.149 | 0.196 | 0.443 | 1/1280 | Moderate symptoms | 186 |
| 51 | M | + | 0.24 | 0.448 | 0.139 | 0.381 | 1/640 | Mild symptoms | 185 |
| 478 | M | + | 0.161 | 0.668 | 0.123 | 0.196 | 1/320 | Mild symptoms | 185 |
| 239 | M | + | 0.29 | 0.991 | 0.251 | 0.239 | 1/320 | Moderate symptoms | 185 |
| 367 | M | + | 0.134 | 0.237 | 0.087 | 0.189 | 1/160 | Mild symptoms | 185 |
| 460 | F | + | 0.098 | 0.511 | 0.075 | 0.09 | 1/1280 | Mild symptoms | 185 |
| 66 | F | + | 0.265 | 0.456 | 0.123 | 0.26 | 1/1280 | Moderate symptoms | 185 |
| 218 | M | + | 0.124 | 0.722 | 0.236 | 0.519 | 1/1280 | Mild symptoms | 185 |
| 287 | F | + | 0.316 | 0.546 | 0.14 | 0.335 | 1/640 | Moderate symptoms | 184 |
| 214 | M | + | 0.117 | 0.638 | 0.175 | 0.251 | 1/640 | Moderate symptoms | 184 |
| 247 | M | + | 0.498 | 0.865 | 0.177 | 0.367 | 1/5120 | Moderate symptoms | 184 |
| 40 | F | + | 0.328 | 0.239 | 0.204 | 0.178 | 1/320 | Moderate symptoms | 184 |
| 262 | M | + | 0.468 | 0.88 | 0.123 | 0.214 | 1/2560 | Moderate symptoms | 184 |
| 258 | M | + | 0.28 | 0.807 | 0.153 | 0.341 | 1/2560 | Moderate symptoms | 184 |
| 453 | F | + | 0.104 | 0.198 | 0.067 | 0.094 | 1/160 | Mild symptoms | 184 |
| 458 | M | + | 0.131 | 0.414 | 0.069 | 0.122 | 1/160 | Mild symptoms | 184 |
| 131 | M | + | 0.172 | 0.468 | 0.125 | 0.274 | 1/160 | Mild symptoms | 184 |
| 217 | M | + | 0.089 | 0.232 | 0.127 | 0.2 | 1/160 | Mild symptoms | 184 |
| 78 | M | + | 0.277 | 0.277 | 0.107 | 0.177 | 1/1280 | Mild symptoms | 184 |
| 61 | F | + | 0.449 | 0.376 | 0.198 | 0.197 | 1/1280 | Moderate symptoms | 184 |
| 279 | M | + | 0.588 | 0.496 | 0.114 | 0.25 | 1/640 | Mild symptoms | 183 |
| 111 | M | + | 0.363 | 0.18 | 0.154 | 0.195 | 1/640 | Mild symptoms | 183 |
| 183 | M | + | 0.102 | 0.405 | 0.179 | 0.302 | 1/640 | Mild symptoms | 183 |
| 113 | M | + | 0.197 | 0.735 | 0.188 | 0.349 | 1/640 | Mild symptoms | 183 |
| 121 | M | + | 0.301 | 0.957 | 0.193 | 0.275 | 1/640 | Moderate symptoms | 183 |
| 22 | F | + | 0.293 | 0.703 | 0.227 | 0.442 | 1/5120 | Mild symptoms | 183 |
| 10 | M | + | 0.36 | 0.649 | 0.132 | 0.36 | 1/320 | Mild symptoms | 183 |
| 456 | F | + | 0.085 | 0.099 | 0.064 | 0.117 | 1/160 | Mild symptoms | 183 |
| 423 | F | + | 0.145 | 0.235 | 0.086 | 0.135 | 1/160 | Moderate symptoms | 183 |
| 147 | M | + | 0.125 | 0.351 | 0.106 | 0.1 | 1/160 | Mild symptoms | 183 |
| 141 | M | + | 0.153 | 0.304 | 0.12 | 0.113 | 1/160 | Mild symptoms | 183 |
| 384 | F | + | 0.164 | 0.389 | 0.12 | 0.185 | 1/160 | Mild symptoms | 183 |
| 389 | M | + | 0.209 | 0.407 | 0.129 | 0.303 | 1/160 | Mild symptoms | 183 |
| 401 | F | + | 0.346 | 0.73 | 0.147 | 0.36 | 1/160 | Mild symptoms | 183 |
| 140 | M | + | 0.157 | 0.274 | 0.151 | 0.143 | 1/160 | Mild symptoms | 183 |
| 153 | M | + | 0.106 | 0.219 | 0.153 | 0.078 | 1/160 | Moderate symptoms | 183 |
| 154 | M | + | 0.088 | 0.231 | 0.184 | 0.112 | 1/160 | Moderate symptoms | 183 |
| 127 | M | + | 0.313 | 0.19 | 0.187 | 0.215 | 1/160 | Mild symptoms | 183 |
| 195 | M | + | 0.183 | 0.283 | 0.32 | 0.518 | 1/160 | Mild symptoms | 183 |
| 96 | M | + | 0.231 | 0.263 | 0.171 | 0.168 | 1/1280 | Moderate symptoms | 183 |
| 92 | M | + | 0.306 | 0.478 | 0.197 | 0.424 | 1/1280 | Moderate symptoms | 183 |
| 202 | M | + | 0.203 | 0.676 | 0.221 | 0.6 | 1/1280 | Mild symptoms | 183 |
| 157 | M | + | 0.105 | 0.242 | 0.213 | 0.13 | 1/640 | Moderate symptoms | 182 |
| 28 | F | + | 0.611 | 0.238 | 0.334 | 0.427 | 1/640 | Mild symptoms | 182 |
| 23 | F | + | 0.257 | 0.599 | 0.185 | 0.5 | 1/5120 | Mild symptoms | 182 |
| 9 | M | + | 0.332 | 0.612 | 0.199 | 0.332 | 1/320 | Mild symptoms | 182 |
| 174 | M | + | 0.121 | 0.374 | 0.271 | 0.099 | 1/320 | Moderate symptoms | 182 |
| 170 | M | + | 0.083 | 0.191 | 0.164 | 0.083 | 1/160 | Moderate symptoms | 182 |
| 138 | M | + | 0.318 | 0.38 | 0.224 | 0.189 | 1/160 | Mild symptoms | 182 |
| 122 | M | + | 0.253 | 0.434 | 0.147 | 0.163 | 1/640 | Moderate symptoms | 181 |
| 168 | M | + | 0.171 | 0.239 | 0.239 | 0.126 | 1/640 | Mild symptoms | 181 |
| 33 | F | + | 0.438 | 0.542 | 0.272 | 0.529 | 1/640 | Mild symptoms | 181 |
| 18 | M | + | 0.261 | 0.407 | 0.318 | 0.666 | 1/640 | Mild symptoms | 181 |
| 2 | M | + | 0.291 | 1.023 | 0.182 | 0.593 | 1/5120 | Mild symptoms | 181 |
| 27 | F | + | 0.289 | 0.217 | 0.159 | 0.492 | 1/320 | Mild symptoms | 181 |
| 222 | M | + | 0.084 | 0.107 | 0.071 | 0.066 | 1/160 | Moderate symptoms | 181 |
| 438 | F | + | 0.152 | 0.688 | 0.077 | 0.353 | 1/160 | Mild symptoms | 181 |
| 477 | F | + | 0.109 | 0.49 | 0.078 | 0.092 | 1/160 | Mild symptoms | 181 |
| 479 | M | + | 0.101 | 0.43 | 0.118 | 0.14 | 1/160 | Mild symptoms | 181 |
| 212 | M | + | 0.078 | 0.318 | 0.128 | 0.194 | 1/160 | Moderate symptoms | 181 |
| 11 | M | + | 0.334 | 0.562 | 0.129 | 0.334 | 1/160 | Mild symptoms | 181 |
| 271 | M | + | 0.307 | 0.865 | 0.139 | 0.288 | 1/1280 | Mild symptoms | 181 |
| 304 | M | + | 0.732 | 0.967 | 0.16 | 0.33 | 1/1280 | Mild symptoms | 181 |
| 12 | M | + | 0.509 | 0.734 | 0.193 | 0.509 | 1/1280 | Mild symptoms | 181 |
| 452 | F | + | 0.156 | 0.521 | 0.076 | 0.551 | 1/640 | Mild symptoms | 180 |
| 19 | M | + | 0.249 | 0.243 | 0.162 | 0.369 | 1/640 | Mild symptoms | 180 |
| 283 | M | + | 0.503 | 1.035 | 0.179 | 0.322 | 1/640 | Moderate symptoms | 180 |
| 233 | M | + | 0.976 | 0.915 | 0.195 | 0.441 | 1/640 | Mild symptoms | 180 |
| 194 | M | + | 0.481 | 0.962 | 0.196 | 0.582 | 1/640 | Moderate symptoms | 180 |
| 29 | F | + | 0.35 | 0.353 | 0.198 | 0.485 | 1/640 | Mild symptoms | 180 |
| 166 | M | + | 0.094 | 0.126 | 0.293 | 0.153 | 1/640 | Mild symptoms | 180 |
| 38 | M | + | 0.406 | 0.542 | 0.292 | 0.652 | 1/5120 | Moderate symptoms | 180 |
| 104 | M | + | 0.182 | 0.248 | 0.123 | 0.187 | 1/320 | Mild symptoms | 180 |
| 475 | F | + | 0.1 | 0.528 | 0.074 | 0.204 | 1/160 | Mild symptoms | 180 |
| 117 | M | + | 0.253 | 0.32 | 0.141 | 0.251 | 1/160 | Mild symptoms | 180 |
| 116 | M | + | 0.28 | 0.326 | 0.173 | 0.307 | 1/160 | Mild symptoms | 180 |
| 251 | M | + | 0.826 | 0.382 | 0.2 | 0.389 | 1/160 | Mild symptoms | 180 |
| 300 | M | + | 0.286 | 0.639 | 0.257 | 0.466 | 1/160 | Mild symptoms | 180 |
| 7 | M | + | 0.431 | 0.336 | 0.281 | 0.191 | 1/160 | Moderate symptoms | 180 |
| 118 | M | + | 0.505 | 0.273 | 0.283 | 0.291 | 1/160 | Mild symptoms | 180 |
| 6 | M | + | 0.454 | 0.398 | 0.326 | 0.323 | 1/160 | Mild symptoms | 180 |
| 20 | M | + | 0.237 | 0.481 | 0.159 | 0.485 | 1/640 | Mild symptoms | 179 |
| 225 | M | + | 0.072 | 0.337 | 0.078 | 0.142 | 1/320 | Mild symptoms | 179 |
| 100 | M | + | 0.166 | 0.423 | 0.125 | 0.192 | 1/320 | Mild symptoms | 179 |
| 415 | F | + | 0.23 | 0.5 | 0.191 | 0.328 | 1/320 | Mild symptoms | 179 |
| 443 | M | + | 0.147 | 0.321 | 0.063 | 0.151 | 1/160 | Moderate symptoms | 179 |
| 462 | F | + | 0.109 | 0.443 | 0.065 | 0.172 | 1/160 | Mild symptoms | 179 |
| 13 | M | + | 0.251 | 0.411 | 0.091 | 0.251 | 1/160 | Mild symptoms | 179 |
| 164 | M | + | 0.072 | 0.079 | 0.129 | 0.082 | 1/160 | Mild symptoms | 179 |
| 30 | F | + | 0.306 | 0.356 | 0.22 | 0.449 | 1/160 | Mild symptoms | 179 |
| 5 | M | + | 0.395 | 0.499 | 0.244 | 0.547 | 1/160 | Moderate symptoms | 179 |
| 80 | M | + | 0.322 | 0.787 | 0.162 | 0.179 | 1/1280 | Mild symptoms | 179 |
| 32 | M | + | 0.297 | 0.315 | 0.217 | 0.434 | 1/640 | Mild symptoms | 178 |
| 1 | M | + | 0.486 | 0.969 | 0.221 | 0.597 | 1/320 | Moderate symptoms | 178 |
| 273 | M | + | 0.464 | 0.503 | 0.282 | 0.505 | 1/320 | Mild symptoms | 178 |
| 368 | M | + | 0.156 | 0.362 | 0.081 | 0.144 | 1/160 | Mild symptoms | 178 |
| 348 | M | + | 0.162 | 0.494 | 0.089 | 0.141 | 1/160 | Moderate symptoms | 178 |
| 15 | M | + | 0.269 | 0.521 | 0.094 | 0.269 | 1/160 | Mild symptoms | 178 |
| 394 | F | + | 0.19 | 0.675 | 0.117 | 0.164 | 1/160 | Mild symptoms | 178 |
| 327 | M | + | 0.44 | 0.887 | 0.119 | 0.388 | 1/160 | Moderate symptoms | 178 |
| 252 | M | + | 0.156 | 0.668 | 0.143 | 0.316 | 1/160 | Mild symptoms | 178 |
| 364 | F | + | 0.175 | 0.563 | 0.153 | 0.599 | 1/160 | Mild symptoms | 178 |
| 4 | M | + | 0.25 | 0.536 | 0.171 | 0.304 | 1/160 | Mild symptoms | 178 |
| 17 | M | + | 0.293 | 0.596 | 0.185 | 0.557 | 1/160 | Mild symptoms | 178 |
| 234 | M | + | 0.074 | 0.105 | 0.219 | 0.489 | 1/160 | Mild symptoms | 178 |
| 73 | F | + | 0.293 | 0.3 | 0.143 | 0.128 | 1/1280 | Mild symptoms | 178 |
| 169 | M | + | 0.108 | 0.425 | 0.205 | 0.104 | 1/1280 | Mild symptoms | 178 |
| 257 | M | + | 0.252 | 0.761 | 0.251 | 0.433 | 1/1280 | Mild symptoms | 178 |
| 196 | M | + | 0.382 | 0.794 | 0.299 | 0.529 | 1/1280 | Mild symptoms | 178 |
| 332 | M | + | 0.154 | 0.357 | 0.093 | 0.131 | 1/640 | Mild symptoms | 177 |
| 45 | F | + | 0.426 | 0.431 | 0.206 | 0.237 | 1/640 | Moderate symptoms | 177 |
| 25 | F | + | 0.314 | 0.417 | 0.214 | 0.42 | 1/640 | Mild symptoms | 177 |
| 21 | M | + | 0.302 | 0.583 | 0.214 | 0.76 | 1/640 | Moderate symptoms | 177 |
| 265 | F | + | 0.31 | 0.866 | 0.239 | 0.49 | 1/5120 | Moderate symptoms | 177 |
| 466 | F | + | 0.109 | 0.559 | 0.07 | 0.127 | 1/160 | Mild symptoms | 177 |
| 226 | M | + | 0.072 | 0.104 | 0.085 | 0.464 | 1/160 | Moderate symptoms | 177 |
| 344 | M | + | 0.223 | 0.648 | 0.109 | 0.312 | 1/160 | Mild symptoms | 177 |
| 67 | F | + | 0.215 | 0.34 | 0.117 | 0.228 | 1/160 | Moderate symptoms | 177 |
| 125 | M | + | 0.351 | 0.359 | 0.207 | 0.265 | 1/160 | Moderate symptoms | 177 |
| 16 | M | + | 0.302 | 0.547 | 0.215 | 0.302 | 1/160 | Mild symptoms | 177 |
| 205 | M | + | 0.244 | 0.806 | 0.209 | 0.279 | 1/1280 | Moderate symptoms | 177 |
| 266 | M | + | 0.819 | 1.162 | 0.389 | 0.814 | 1/5120 | Moderate symptoms | 176 |
| 441 | M | + | 0.119 | 0.48 | 0.071 | 0.398 | 1/320 | Mild symptoms | 176 |
| 412 | F | + | 0.156 | 0.164 | 0.095 | 0.323 | 1/320 | Mild symptoms | 176 |
| 272 | M | + | 0.191 | 0.612 | 0.12 | 0.279 | 1/320 | Mild symptoms | 176 |
| 193 | M | + | 0.122 | 0.253 | 0.224 | 0.342 | 1/320 | Moderate symptoms | 176 |
| 284 | M | + | 0.533 | 1.055 | 0.172 | 0.314 | 1/2560 | Mild symptoms | 176 |
| 206 | M | + | 0.187 | 0.413 | 0.216 | 0.18 | 1/1280 | Mild symptoms | 176 |
| 315 | F | + | 0.335 | 0.82 | 0.226 | 0.736 | 1/1280 | Moderate symptoms | 176 |
| 24 | F | + | 0.306 | 0.616 | 0.237 | 0.854 | 1/1280 | Mild symptoms | 176 |
| 267 | F | + | 0.255 | 0.753 | 0.113 | 0.256 | 1/640 | Moderate symptoms | 175 |
| 53 | F | + | 0.217 | 0.484 | 0.139 | 0.128 | 1/640 | Mild symptoms | 175 |
| 342 | M | + | 0.178 | 0.876 | 0.221 | 0.416 | 1/320 | Mild symptoms | 175 |
| 105 | M | + | 0.352 | 1.036 | 0.242 | 0.432 | 1/320 | Mild symptoms | 175 |
| 237 | M | + | 0.316 | 0.411 | 0.58 | 0.791 | 1/320 | Mild symptoms | 175 |
| 3 | M | + | 0.3 | 1 | 0.168 | 0.478 | 1/2560 | Mild symptoms | 175 |
| 399 | F | + | 0.116 | 0.125 | 0.079 | 0.09 | 1/160 | Mild symptoms | 175 |
| 14 | M | + | 0.29 | 0.453 | 0.083 | 0.29 | 1/160 | Moderate symptoms | 175 |
| 404 | M | + | 0.233 | 0.382 | 0.105 | 0.145 | 1/160 | Moderate symptoms | 175 |
| 46 | F | + | 0.262 | 0.411 | 0.154 | 0.225 | 1/160 | Mild symptoms | 175 |
| 363 | F | + | 0.205 | 0.782 | 0.105 | 0.276 | 1/1280 | Mild symptoms | 175 |
| 71 | F | + | 0.238 | 0.308 | 0.107 | 0.162 | 1/1280 | Mild symptoms | 175 |
| 79 | M | + | 0.201 | 0.71 | 0.114 | 0.228 | 1/1280 | Moderate symptoms | 175 |
| 26 | F | + | 0.297 | 0.458 | 0.262 | 0.514 | 1/1280 | Mild symptoms | 175 |
| 230 | M | + | 0.393 | 0.782 | 0.149 | 0.326 | 1/640 | Moderate symptoms | 174 |
| 305 | M | + | 0.294 | 0.857 | 0.28 | 0.536 | 1/640 | Mild symptoms | 174 |
| 476 | M | + | 0.11 | 0.109 | 0.077 | 0.071 | 1/320 | Mild symptoms | 174 |
| 290 | M | + | 0.17 | 0.956 | 0.139 | 0.272 | 1/320 | Mild symptoms | 174 |
| 64 | M | + | 0.316 | 0.51 | 0.163 | 0.178 | 1/320 | Moderate symptoms | 174 |
| 454 | M | + | 0.1 | 0.519 | 0.075 | 0.184 | 1/160 | Mild symptoms | 174 |
| 380 | M | + | 0.151 | 0.541 | 0.094 | 0.253 | 1/160 | Mild symptoms | 174 |
| 362 | M | + | 0.134 | 0.573 | 0.096 | 0.462 | 1/160 | Moderate symptoms | 174 |
| 352 | M | + | 0.37 | 0.489 | 0.105 | 0.251 | 1/160 | Moderate symptoms | 174 |
| 427 | M | + | 0.213 | 0.247 | 0.134 | 0.315 | 1/160 | Moderate symptoms | 174 |
| 129 | M | + | 0.228 | 0.377 | 0.163 | 0.41 | 1/160 | Moderate symptoms | 174 |
| 63 | M | + | 0.279 | 0.521 | 0.153 | 0.165 | 1/1280 | Moderate symptoms | 174 |
| 208 | M | + | 0.128 | 0.425 | 0.161 | 0.274 | 1/1280 | Moderate symptoms | 174 |
| 99 | M | + | 0.276 | 0.802 | 0.204 | 0.77 | 1/1280 | Moderate symptoms | 174 |
| 176 | M | + | 0.126 | 0.501 | 0.261 | 0.103 | 1/1280 | Moderate symptoms | 174 |
| 461 | F | + | 0.085 | 0.404 | 0.07 | 0.121 | 1/640 | Mild symptoms | 173 |
| 68 | F | + | 0.363 | 0.359 | 0.172 | 0.257 | 1/5120 | Mild symptoms | 173 |
| 333 | F | + | 0.146 | 0.539 | 0.124 | 0.775 | 1/320 | Moderate symptoms | 173 |
| 289 | F | + | 0.2 | 0.876 | 0.173 | 0.389 | 1/320 | Mild symptoms | 173 |
| 97 | M | + | 0.342 | 0.643 | 0.187 | 0.379 | 1/320 | Mild symptoms | 173 |
| 310 | F | + | 0.393 | 0.758 | 0.373 | 0.727 | 1/320 | Mild symptoms | 173 |
| 276 | M | + | 0.281 | 1.073 | 0.122 | 0.307 | 1/2560 | Mild symptoms | 173 |
| 223 | M | + | 0.068 | 0.335 | 0.071 | 0.076 | 1/160 | Mild symptoms | 173 |
| 431 | F | + | 0.155 | 0.229 | 0.081 | 0.507 | 1/160 | Mild symptoms | 173 |
| 143 | M | + | 0.215 | 0.466 | 0.151 | 0.182 | 1/160 | Mild symptoms | 173 |
| 261 | F | + | 0.125 | 0.394 | 0.154 | 0.426 | 1/160 | Moderate symptoms | 173 |
| 180 | M | + | 0.108 | 0.188 | 0.196 | 0.294 | 1/160 | Moderate symptoms | 173 |
| 8 | M | + | 0.364 | 0.266 | 0.28 | 0.202 | 1/160 | Mild symptoms | 173 |
| 55 | F | + | 0.339 | 0.402 | 0.202 | 0.674 | 1/1280 | Mild symptoms | 173 |
| 90 | M | + | 0.338 | 0.936 | 0.205 | 0.369 | 1/1280 | Mild symptoms | 173 |
| 385 | F | + | 0.205 | 0.713 | 0.129 | 0.259 | 1/640 | Mild symptoms | 172 |
| 115 | M | + | 0.378 | 0.671 | 0.189 | 0.269 | 1/640 | Mild symptoms | 172 |
| 227 | M | + | 0.136 | 0.35 | 0.316 | 0.733 | 1/640 | Mild symptoms | 172 |
| 69 | F | + | 0.265 | 0.331 | 0.12 | 0.197 | 1/5120 | Mild symptoms | 172 |
| 77 | F | + | 0.288 | 0.356 | 0.132 | 0.167 | 1/5120 | Mild symptoms | 172 |
| 328 | F | + | 0.436 | 0.88 | 0.117 | 0.376 | 1/320 | Mild symptoms | 172 |
| 292 | M | + | 0.319 | 0.808 | 0.136 | 0.319 | 1/320 | Moderate symptoms | 172 |
| 163 | M | + | 0.105 | 0.539 | 0.152 | 0.196 | 1/320 | Moderate symptoms | 172 |
| 81 | F | + | 0.226 | 0.622 | 0.157 | 0.601 | 1/2560 | Mild symptoms | 172 |
| 391 | F | + | 0.099 | 0.324 | 0.076 | 0.101 | 1/160 | Mild symptoms | 172 |
| 132 | M | + | 0.204 | 0.397 | 0.147 | 0.189 | 1/160 | Mild symptoms | 172 |
| 198 | M | + | 0.12 | 0.19 | 0.156 | 0.225 | 1/160 | Mild symptoms | 172 |
| 210 | M | + | 0.093 | 0.213 | 0.169 | 0.145 | 1/160 | Moderate symptoms | 172 |
| 145 | M | + | 0.272 | 0.184 | 0.196 | 0.157 | 1/160 | Moderate symptoms | 172 |
| 72 | M | + | 0.236 | 0.672 | 0.113 | 0.219 | 1/1280 | Mild symptoms | 172 |
| 238 | M | + | 0.679 | 1.08 | 0.199 | 0.488 | 1/1280 | Mild symptoms | 172 |
| 259 | M | + | 0.299 | 0.969 | 0.232 | 0.538 | 1/1280 | Moderate symptoms | 172 |
| 470 | M | + | 0.174 | 0.569 | 0.08 | 0.32 | 1/320 | Mild symptoms | 171 |
| 191 | M | + | 0.084 | 0.207 | 0.212 | 0.188 | 1/320 | Mild symptoms | 171 |
| 439 | M | + | 0.143 | 0.369 | 0.058 | 0.076 | 1/160 | Mild symptoms | 171 |
| 444 | M | + | 0.147 | 0.549 | 0.063 | 0.175 | 1/160 | Mild symptoms | 171 |
| 446 | M | + | 0.136 | 0.139 | 0.071 | 0.083 | 1/160 | Mild symptoms | 171 |
| 228 | M | + | 0.069 | 0.122 | 0.107 | 0.177 | 1/160 | Moderate symptoms | 171 |
| 377 | M | + | 0.223 | 0.501 | 0.128 | 0.125 | 1/160 | Mild symptoms | 171 |
| 425 | M | + | 0.461 | 0.582 | 0.228 | 0.197 | 1/160 | Mild symptoms | 171 |
| 277 | M | + | 0.317 | 1.017 | 0.172 | 0.599 | 1/1280 | Moderate symptoms | 171 |
| 307 | M | + | 0.456 | 0.828 | 0.259 | 0.705 | 1/1280 | Moderate symptoms | 171 |
| 450 | M | + | 0.133 | 0.532 | 0.08 | 0.127 | 1/320 | Mild symptoms | 170 |
| 91 | M | + | 0.595 | 0.906 | 0.209 | 0.377 | 1/2560 | Moderate symptoms | 170 |
| 435 | M | + | 0.175 | 0.656 | 0.093 | 0.316 | 1/160 | Mild symptoms | 170 |
| 387 | F | + | 0.175 | 0.43 | 0.134 | 0.476 | 1/160 | Mild symptoms | 170 |
| 162 | M | + | 0.079 | 0.095 | 0.187 | 0.099 | 1/160 | Mild symptoms | 170 |
| 343 | M | + | 0.288 | 0.756 | 0.134 | 0.263 | 1/1280 | Moderate symptoms | 170 |
| 207 | M | + | 0.125 | 0.598 | 0.16 | 0.156 | 1/1280 | Moderate symptoms | 170 |
| 95 | M | + | 0.257 | 0.231 | 0.209 | 0.298 | 1/1280 | Mild symptoms | 170 |
| 213 | M | + | 0.136 | 0.571 | 0.278 | 0.288 | 1/1280 | Moderate symptoms | 170 |
| 76 | F | + | 0.232 | 0.281 | 0.117 | 0.147 | 1/5120 | Mild symptoms | 169 |
| 242 | M | + | 0.252 | 0.634 | 0.161 | 0.516 | 1/5120 | Mild symptoms | 169 |
| 250 | M | + | 0.279 | 1.076 | 0.227 | 0.525 | 1/5120 | Mild symptoms | 169 |
| 433 | F | + | 0.222 | 0.625 | 0.082 | 0.183 | 1/320 | Mild symptoms | 169 |
| 128 | M | + | 0.316 | 0.219 | 0.171 | 0.31 | 1/320 | Mild symptoms | 169 |
| 317 | F | + | 0.278 | 0.547 | 0.202 | 0.499 | 1/320 | Moderate symptoms | 169 |
| 376 | M | + | 0.108 | 0.359 | 0.071 | 0.118 | 1/160 | Mild symptoms | 169 |
| 220 | M | + | 0.067 | 0.193 | 0.078 | 0.112 | 1/160 | Mild symptoms | 169 |
| 428 | M | + | 0.111 | 0.326 | 0.086 | 0.126 | 1/160 | Moderate symptoms | 169 |
| 360 | M | + | 0.203 | 0.379 | 0.087 | 0.247 | 1/160 | Mild symptoms | 169 |
| 480 | M | + | 0.119 | 0.438 | 0.124 | 0.185 | 1/160 | Mild symptoms | 169 |
| 159 | M | + | 0.079 | 0.077 | 0.178 | 0.081 | 1/160 | Moderate symptoms | 169 |
| 188 | M | + | 0.126 | 0.39 | 0.438 | 0.315 | 1/160 | Mild symptoms | 169 |
| 119 | M | + | 0.225 | 0.248 | 0.128 | 0.247 | 1/640 | Mild symptoms | 168 |
| 93 | M | + | 0.17 | 0.402 | 0.137 | 0.228 | 1/640 | Moderate symptoms | 168 |
| 326 | M | + | 0.442 | 0.893 | 0.156 | 0.506 | 1/640 | Mild symptoms | 168 |
| 286 | F | + | 0.664 | 0.891 | 0.172 | 0.415 | 1/640 | Moderate symptoms | 168 |
| 319 | M | + | 0.766 | 0.827 | 0.174 | 0.354 | 1/640 | Mild symptoms | 168 |
| 52 | M | + | 0.492 | 0.582 | 0.261 | 0.237 | 1/640 | Mild symptoms | 168 |
| 185 | M | + | 0.231 | 0.636 | 0.309 | 0.454 | 1/640 | Mild symptoms | 168 |
| 181 | M | + | 0.115 | 0.239 | 0.186 | 0.251 | 1/320 | Moderate symptoms | 168 |
| 293 | M | + | 0.199 | 0.872 | 0.191 | 0.3 | 1/320 | Moderate symptoms | 168 |
| 372 | M | + | 0.096 | 0.303 | 0.073 | 0.126 | 1/160 | Mild symptoms | 168 |
| 400 | F | + | 0.107 | 0.116 | 0.099 | 0.097 | 1/160 | Moderate symptoms | 168 |
| 351 | F | + | 0.234 | 0.588 | 0.127 | 0.114 | 1/160 | Moderate symptoms | 168 |
| 314 | F | + | 0.516 | 0.308 | 0.247 | 0.567 | 1/160 | Moderate symptoms | 168 |
| 126 | M | + | 0.552 | 0.33 | 0.332 | 0.246 | 1/160 | Mild symptoms | 168 |
| 448 | M | + | 0.154 | 0.656 | 0.066 | 0.176 | 1/1280 | Mild symptoms | 168 |
| 395 | F | + | 0.661 | 0.543 | 0.127 | 0.197 | 1/640 | Mild symptoms | 167 |
| 324 | M | + | 0.449 | 0.901 | 0.189 | 0.535 | 1/640 | Mild symptoms | 167 |
| 421 | F | + | 0.15 | 0.356 | 0.08 | 0.138 | 1/160 | Moderate symptoms | 167 |
| 74 | M | + | 0.273 | 0.314 | 0.135 | 0.251 | 1/160 | Mild symptoms | 167 |
| 108 | M | + | 0.323 | 0.36 | 0.158 | 0.306 | 1/160 | Mild symptoms | 167 |
| 313 | F | + | 0.452 | 0.61 | 0.203 | 0.609 | 1/160 | Mild symptoms | 167 |
| 204 | M | + | 0.14 | 0.452 | 0.173 | 0.429 | 1/1280 | Moderate symptoms | 167 |
| 59 | M | + | 0.408 | 0.405 | 0.179 | 0.214 | 1/1280 | Mild symptoms | 167 |
| 411 | M | + | 0.145 | 0.621 | 0.097 | 0.237 | 1/640 | Mild symptoms | 166 |
| 463 | F | + | 0.178 | 0.646 | 0.128 | 0.142 | 1/640 | Mild symptoms | 166 |
| 58 | F | + | 0.392 | 0.408 | 0.175 | 0.274 | 1/640 | Mild symptoms | 166 |
| 165 | M | + | 0.148 | 0.709 | 0.22 | 0.271 | 1/5120 | Mild symptoms | 166 |
| 430 | M | + | 0.176 | 0.561 | 0.081 | 0.306 | 1/320 | Mild symptoms | 166 |
| 357 | F | + | 0.122 | 0.298 | 0.083 | 0.152 | 1/320 | Mild symptoms | 166 |
| 346 | F | + | 0.157 | 0.472 | 0.119 | 0.19 | 1/320 | Moderate symptoms | 166 |
| 345 | M | + | 0.201 | 0.511 | 0.12 | 0.289 | 1/320 | Mild symptoms | 166 |
| 373 | M | + | 0.177 | 0.331 | 0.087 | 0.133 | 1/2560 | Mild symptoms | 166 |
| 457 | F | + | 0.1 | 0.123 | 0.114 | 0.078 | 1/160 | Mild symptoms | 166 |
| 417 | M | + | 0.127 | 0.151 | 0.09 | 0.154 | 1/640 | Mild symptoms | 165 |
| 278 | M | + | 0.606 | 0.802 | 0.118 | 0.365 | 1/640 | Mild symptoms | 165 |
| 110 | M | + | 0.388 | 0.164 | 0.182 | 0.299 | 1/640 | Moderate symptoms | 165 |
| 200 | M | + | 0.196 | 0.568 | 0.191 | 0.317 | 1/640 | Mild symptoms | 165 |
| 190 | M | + | 0.214 | 0.679 | 0.302 | 0.407 | 1/640 | Mild symptoms | 165 |
| 270 | M | + | 0.296 | 1.003 | 0.21 | 0.38 | 1/5120 | Mild symptoms | 165 |
| 254 | M | + | 0.233 | 0.824 | 0.22 | 0.29 | 1/5120 | Mild symptoms | 165 |
| 296 | M | + | 0.666 | 1.009 | 0.274 | 0.422 | 1/320 | Mild symptoms | 165 |
| 86 | M | + | 0.194 | 0.415 | 0.136 | 0.242 | 1/2560 | Moderate symptoms | 165 |
| 436 | M | + | 0.126 | 0.499 | 0.077 | 0.382 | 1/160 | Mild symptoms | 165 |
| 402 | F | + | 0.121 | 0.101 | 0.08 | 0.092 | 1/160 | Mild symptoms | 165 |
| 403 | M | + | 0.185 | 0.156 | 0.084 | 0.093 | 1/160 | Mild symptoms | 165 |
| 54 | M | + | 0.209 | 0.395 | 0.131 | 0.159 | 1/160 | Mild symptoms | 165 |
| 381 | F | + | 0.45 | 0.477 | 0.329 | 0.267 | 1/160 | Mild symptoms | 165 |
| 219 | M | + | 0.098 | 0.885 | 0.104 | 0.582 | 1/1280 | Mild symptoms | 165 |
| 246 | M | + | 0.317 | 0.764 | 0.197 | 0.433 | 1/5120 | Mild symptoms | 164 |
| 410 | F | + | 0.174 | 0.397 | 0.111 | 0.432 | 1/320 | Mild symptoms | 164 |
| 112 | M | + | 0.413 | 0.299 | 0.229 | 0.247 | 1/320 | Mild symptoms | 164 |
| 148 | M | + | 0.259 | 0.5 | 0.256 | 0.279 | 1/320 | Moderate symptoms | 164 |
| 264 | M | + | 0.277 | 0.806 | 0.189 | 0.467 | 1/2560 | Moderate symptoms | 164 |
| 186 | M | + | 0.485 | 0.968 | 0.207 | 0.585 | 1/2560 | Mild symptoms | 164 |
| 361 | M | + | 0.141 | 0.183 | 0.09 | 0.105 | 1/160 | Moderate symptoms | 164 |
| 120 | M | + | 0.359 | 0.196 | 0.175 | 0.221 | 1/160 | Moderate symptoms | 164 |
| 133 | M | + | 0.344 | 0.303 | 0.236 | 0.189 | 1/160 | Mild symptoms | 164 |
| 178 | M | + | 0.142 | 0.198 | 0.255 | 0.227 | 1/1280 | Moderate symptoms | 164 |
| 88 | M | + | 0.282 | 0.357 | 0.181 | 0.24 | 1/640 | Mild symptoms | 163 |
| 41 | F | + | 0.432 | 0.739 | 0.24 | 0.552 | 1/640 | Mild symptoms | 163 |
| 36 | M | + | 0.39 | 0.404 | 0.285 | 0.724 | 1/640 | Mild symptoms | 163 |
| 75 | M | + | 0.238 | 0.733 | 0.129 | 0.551 | 1/5120 | Mild symptoms | 163 |
| 134 | F | + | 0.319 | 0.369 | 0.215 | 0.207 | 1/320 | Mild symptoms | 163 |
| 335 | M | + | 0.423 | 0.855 | 0.105 | 0.368 | 1/2560 | Moderate symptoms | 163 |
| 84 | M | + | 0.27 | 0.621 | 0.174 | 0.487 | 1/2560 | Mild symptoms | 163 |
| 224 | M | + | 0.125 | 0.152 | 0.07 | 0.118 | 1/160 | Mild symptoms | 163 |
| 378 | F | + | 0.156 | 0.415 | 0.101 | 0.157 | 1/160 | Mild symptoms | 163 |
| 354 | F | + | 0.192 | 0.245 | 0.106 | 0.116 | 1/160 | Moderate symptoms | 163 |
| 274 | M | + | 0.27 | 0.712 | 0.136 | 0.378 | 1/160 | Mild symptoms | 163 |
| 355 | M | + | 0.345 | 0.432 | 0.141 | 0.36 | 1/160 | Mild symptoms | 163 |
| 285 | M | + | 0.525 | 0.609 | 0.141 | 0.555 | 1/1280 | Moderate symptoms | 163 |
| 89 | M | + | 0.243 | 0.817 | 0.143 | 0.353 | 1/1280 | Moderate symptoms | 163 |
| 82 | F | + | 0.231 | 0.341 | 0.186 | 0.165 | 1/1280 | Mild symptoms | 163 |
| 325 | M | + | 0.443 | 0.897 | 0.186 | 0.525 | 1/1280 | Moderate symptoms | 163 |
| 330 | M | + | 0.257 | 0.849 | 0.189 | 0.126 | 1/1280 | Moderate symptoms | 163 |
| 231 | M | + | 0.474 | 0.945 | 0.194 | 0.558 | 1/1280 | Mild symptoms | 163 |
| 240 | M | + | 0.324 | 0.982 | 0.277 | 0.584 | 1/1280 | Mild symptoms | 163 |
| 44 | M | + | 0.324 | 0.684 | 0.15 | 0.408 | 1/640 | Mild symptoms | 162 |
| 232 | M | + | 0.222 | 0.561 | 0.412 | 0.803 | 1/640 | Mild symptoms | 162 |
| 474 | F | + | 0.07 | 0.082 | 0.068 | 0.065 | 1/320 | Mild symptoms | 162 |
| 130 | M | + | 0.206 | 0.418 | 0.132 | 0.15 | 1/320 | Mild symptoms | 162 |
| 473 | M | + | 0.082 | 0.263 | 0.068 | 0.067 | 1/160 | Mild symptoms | 162 |
| 471 | F | + | 0.137 | 0.349 | 0.078 | 0.349 | 1/160 | Moderate symptoms | 162 |
| 371 | F | + | 0.108 | 0.292 | 0.081 | 0.127 | 1/160 | Mild symptoms | 162 |
| 445 | F | + | 0.14 | 0.432 | 0.086 | 0.326 | 1/160 | Mild symptoms | 162 |
| 142 | M | + | 0.165 | 0.255 | 0.122 | 0.123 | 1/160 | Moderate symptoms | 162 |
| 379 | F | + | 0.296 | 0.26 | 0.124 | 0.136 | 1/160 | Mild symptoms | 162 |
| 173 | M | + | 0.075 | 0.069 | 0.16 | 0.081 | 1/160 | Mild symptoms | 162 |
| 102 | M | + | 0.246 | 0.205 | 0.169 | 0.199 | 1/160 | Moderate symptoms | 162 |
| 172 | M | + | 0.106 | 0.137 | 0.184 | 0.082 | 1/160 | Mild symptoms | 162 |
| 275 | M | + | 0.295 | 0.349 | 0.213 | 0.492 | 1/160 | Moderate symptoms | 162 |
| 447 | M | + | 0.123 | 0.471 | 0.064 | 0.341 | 1/640 | Mild symptoms | 161 |
| 215 | M | + | 0.112 | 0.638 | 0.137 | 0.195 | 1/640 | Mild symptoms | 161 |
| 35 | F | + | 0.237 | 0.425 | 0.207 | 0.427 | 1/640 | Mild symptoms | 161 |
| 299 | M | + | 0.165 | 0.982 | 0.227 | 0.456 | 1/640 | Moderate symptoms | 161 |
| 374 | F | + | 0.116 | 0.374 | 0.076 | 0.245 | 1/320 | Mild symptoms | 161 |
| 409 | M | + | 0.139 | 0.346 | 0.093 | 0.149 | 1/320 | Moderate symptoms | 161 |
| 359 | M | + | 0.195 | 0.689 | 0.112 | 0.227 | 1/320 | Mild symptoms | 161 |
| 236 | M | + | 0.698 | 0.996 | 0.134 | 0.294 | 1/320 | Mild symptoms | 161 |
| 98 | M | + | 0.215 | 0.614 | 0.151 | 0.293 | 1/2560 | Mild symptoms | 161 |
| 260 | M | + | 0.354 | 0.895 | 0.154 | 0.332 | 1/2560 | Mild symptoms | 161 |
| 437 | F | + | 0.102 | 0.29 | 0.068 | 0.102 | 1/160 | Mild symptoms | 161 |
| 397 | F | + | 0.203 | 0.497 | 0.091 | 0.194 | 1/160 | Mild symptoms | 161 |
| 311 | M | + | 0.142 | 0.486 | 0.133 | 0.354 | 1/160 | Mild symptoms | 161 |
| 192 | M | + | 0.126 | 0.173 | 0.139 | 0.187 | 1/160 | Moderate symptoms | 161 |
| 321 | F | + | 0.455 | 0.735 | 0.185 | 0.423 | 1/160 | Mild symptoms | 161 |
| 303 | F | + | 0.151 | 0.96 | 0.161 | 0.349 | 1/1280 | Mild symptoms | 161 |
| 323 | M | + | 0.367 | 0.977 | 0.189 | 0.391 | 1/1280 | Mild symptoms | 161 |
| 85 | M | + | 0.358 | 0.492 | 0.206 | 0.256 | 1/1280 | Mild symptoms | 161 |
| 197 | M | + | 0.153 | 0.595 | 0.16 | 0.381 | 1/640 | Mild symptoms | 160 |
| 459 | M | + | 0.076 | 0.478 | 0.074 | 0.152 | 1/320 | Mild symptoms | 160 |
| 416 | M | + | 0.159 | 0.435 | 0.096 | 0.35 | 1/160 | Mild symptoms | 160 |
| 366 | F | + | 0.188 | 0.386 | 0.11 | 0.142 | 1/160 | Mild symptoms | 160 |
| 158 | M | + | 0.067 | 0.266 | 0.125 | 0.077 | 1/160 | Moderate symptoms | 160 |
| 160 | M | + | 0.087 | 0.081 | 0.186 | 0.077 | 1/160 | Moderate symptoms | 160 |
| 109 | M | + | 0.29 | 0.402 | 0.22 | 0.307 | 1/1280 | Mild symptoms | 160 |
| 56 | F | + | 0.472 | 0.361 | 0.247 | 0.378 | 1/1280 | Mild symptoms | 160 |
| 339 | M | + | 0.141 | 0.713 | 0.099 | 0.493 | 1/640 | Mild symptoms | 159 |
| 484 | F | + | 0.135 | 0.693 | 0.109 | 0.127 | 1/640 | Mild symptoms | 159 |
| 43 | F | + | 0.487 | 0.666 | 0.269 | 0.449 | 1/640 | Moderate symptoms | 159 |
| 70 | M | + | 0.324 | 0.713 | 0.143 | 0.238 | 1/5120 | Mild symptoms | 159 |
| 244 | M | + | 0.424 | 0.808 | 0.176 | 0.386 | 1/5120 | Moderate symptoms | 159 |
| 255 | M | + | 0.463 | 0.924 | 0.19 | 0.545 | 1/5120 | Mild symptoms | 159 |
| 42 | F | + | 0.232 | 0.298 | 0.138 | 0.163 | 1/320 | Mild symptoms | 159 |
| 294 | M | + | 0.198 | 1.126 | 0.145 | 0.393 | 1/320 | Moderate symptoms | 159 |
| 263 | F | + | 0.437 | 1.053 | 0.223 | 0.559 | 1/2560 | Moderate symptoms | 159 |
| 39 | M | + | 0.289 | 0.46 | 0.24 | 0.579 | 1/2560 | Mild symptoms | 159 |
| 482 | F | + | 0.108 | 0.174 | 0.114 | 0.118 | 1/160 | Moderate symptoms | 159 |
| 481 | M | + | 0.12 | 0.363 | 0.124 | 0.129 | 1/160 | Mild symptoms | 159 |
| 60 | M | + | 0.373 | 0.779 | 0.17 | 0.282 | 1/1280 | Mild symptoms | 159 |
| 184 | M | + | 0.207 | 0.603 | 0.29 | 0.316 | 1/1280 | Mild symptoms | 159 |
| 434 | M | + | 0.121 | 0.179 | 0.066 | 0.07 | 1/320 | Mild symptoms | 158 |
| 468 | M | + | 0.111 | 0.348 | 0.071 | 0.093 | 1/160 | Mild symptoms | 158 |
| 413 | F | + | 0.105 | 0.118 | 0.08 | 0.145 | 1/160 | Mild symptoms | 158 |
| 347 | M | + | 0.223 | 0.174 | 0.127 | 0.1 | 1/160 | Mild symptoms | 158 |
| 103 | M | + | 0.461 | 0.183 | 0.315 | 0.259 | 1/160 | Moderate symptoms | 158 |
| 221 | M | + | 0.088 | 0.551 | 0.073 | 0.134 | 1/1280 | Mild symptoms | 158 |
| 297 | F | + | 0.344 | 1.114 | 0.117 | 0.319 | 1/1280 | Mild symptoms | 158 |
| 337 | M | + | 0.185 | 0.59 | 0.104 | 0.236 | 1/640 | Mild symptoms | 157 |
| 306 | F | + | 0.345 | 0.848 | 0.186 | 0.542 | 1/640 | Mild symptoms | 157 |
| 405 | F | + | 0.124 | 0.723 | 0.103 | 0.339 | 1/320 | Moderate symptoms | 157 |
| 422 | F | + | 0.191 | 0.261 | 0.115 | 0.162 | 1/160 | Mild symptoms | 157 |
| 203 | M | + | 0.095 | 0.23 | 0.129 | 0.433 | 1/160 | Mild symptoms | 157 |
| 161 | M | + | 0.082 | 0.083 | 0.193 | 0.09 | 1/160 | Mild symptoms | 157 |
| 268 | M | + | 0.214 | 0.837 | 0.165 | 0.351 | 1/1280 | Mild symptoms | 157 |
| 398 | M | + | 0.127 | 0.11 | 0.09 | 0.081 | 1/640 | Mild symptoms | 156 |
| 209 | M | + | 0.119 | 0.753 | 0.165 | 0.219 | 1/640 | Moderate symptoms | 156 |
| 241 | M | + | 0.92 | 1.048 | 0.141 | 0.252 | 1/5120 | Moderate symptoms | 156 |
| 37 | M | + | 0.326 | 0.425 | 0.246 | 0.471 | 1/5120 | Moderate symptoms | 156 |
| 34 | F | + | 0.285 | 0.458 | 0.185 | 0.572 | 1/320 | Mild symptoms | 156 |
| 152 | M | + | 0.357 | 0.461 | 0.219 | 0.353 | 1/320 | Moderate symptoms | 156 |
| 31 | M | + | 0.478 | 0.345 | 0.324 | 0.521 | 1/320 | Mild symptoms | 156 |
| 280 | F | + | 0.263 | 0.921 | 0.203 | 0.491 | 1/2560 | Moderate symptoms | 156 |
| 414 | F | + | 0.156 | 0.129 | 0.107 | 0.095 | 1/160 | Mild symptoms | 156 |
| 322 | F | + | 0.499 | 0.792 | 0.152 | 0.411 | 1/160 | Mild symptoms | 156 |
| 396 | M | + | 0.235 | 0.682 | 0.092 | 0.376 | 1/320 | Mild symptoms | 155 |
| 182 | M | + | 0.084 | 0.126 | 0.167 | 0.268 | 1/320 | Mild symptoms | 155 |
| 429 | F | + | 0.165 | 0.111 | 0.081 | 0.098 | 1/160 | Mild symptoms | 155 |
| 388 | M | + | 0.139 | 0.367 | 0.084 | 0.202 | 1/160 | Mild symptoms | 155 |
| 144 | M | + | 0.303 | 0.3 | 0.17 | 0.146 | 1/160 | Mild symptoms | 155 |
| 464 | F | + | 0.086 | 0.158 | 0.066 | 0.09 | 1/1280 | Mild symptoms | 155 |
| 211 | M | + | 0.146 | 0.901 | 0.146 | 0.404 | 1/5120 | Mild symptoms | 154 |
| 418 | F | + | 0.112 | 0.116 | 0.086 | 0.108 | 1/160 | Mild symptoms | 154 |
| 139 | M | + | 0.219 | 0.265 | 0.14 | 0.13 | 1/160 | Moderate symptoms | 154 |
| 101 | M | + | 0.287 | 0.475 | 0.193 | 0.253 | 1/160 | Moderate symptoms | 154 |
| 253 | M | + | 0.47 | 0.944 | 0.193 | 0.556 | 1/1280 | Mild symptoms | 154 |
