# Supplementary material for: Potent and Persistent Antibody Response in COVID-19 Recovered Patients
Source: Front Immunol. 2021 May 28;12:659041. doi: 10.3389/fimmu.2021.659041 (PMC8193946; doi:10.3389/fimmu.2021.659041)
Supplement: Supplementary file 2 [file Table_2.doc]

**Supplemental Table 2. The characteristics of negative controls in this study**

| **NO.** | **Sex** | **Age** | **S-IgM (OD450) (1:400)** | **S-IgG (OD450) (1:400)** | **N-IgM (OD450) (1:400)** | **N-IgG (OD450) (1:400)** |
| --- | --- | --- | --- | --- | --- | --- |
| 1 | M | 64 | 0.127 | 0.058 | 0.089 | 0.06 |
| 2 | M | 42 | 0.139 | 0.121 | 0.071 | 0.136 |
| 3 | F | 47 | 0.096 | 0.067 | 0.095 | 0.141 |
| 4 | F | 45 | 0.077 | 0.127 | 0.135 | 0.057 |
| 5 | M | 33 | 0.112 | 0.066 | 0.055 | 0.063 |
| 6 | F | 31 | 0.154 | 0.063 | 0.119 | 0.092 |
| 7 | M | 27 | 0.055 | 0.124 | 0.114 | 0.114 |
| 8 | F | 25 | 0.08 | 0.134 | 0.07 | 0.135 |
| 9 | F | 41 | 0.066 | 0.055 | 0.129 | 0.121 |
| 10 | F | 28 | 0.131 | 0.112 | 0.149 | 0.072 |
| 11 | M | 30 | 0.118 | 0.117 | 0.087 | 0.126 |
| 12 | M | 32 | 0.125 | 0.115 | 0.061 | 0.063 |
| 13 | M | 25 | 0.154 | 0.081 | 0.066 | 0.105 |
| 14 | F | 40 | 0.104 | 0.1 | 0.063 | 0.1 |
| 15 | M | 37 | 0.089 | 0.123 | 0.137 | 0.116 |
| 16 | M | 41 | 0.065 | 0.064 | 0.101 | 0.111 |
| 17 | F | 28 | 0.114 | 0.077 | 0.112 | 0.141 |
| 18 | M | 28 | 0.085 | 0.113 | 0.153 | 0.134 |
| 19 | F | 56 | 0.152 | 0.107 | 0.071 | 0.082 |
| 20 | F | 24 | 0.112 | 0.132 | 0.123 | 0.106 |
| 21 | F | 25 | 0.066 | 0.067 | 0.055 | 0.111 |
| 22 | F | 25 | 0.133 | 0.09 | 0.094 | 0.094 |
| 23 | M | 24 | 0.09 | 0.105 | 0.121 | 0.137 |
| 24 | F | 30 | 0.106 | 0.094 | 0.071 | 0.126 |
| 25 | F | 43 | 0.055 | 0.094 | 0.124 | 0.109 |
| 26 | M | 37 | 0.078 | 0.09 | 0.144 | 0.06 |
| 27 | F | 41 | 0.056 | 0.132 | 0.084 | 0.151 |
| 28 | M | 66 | 0.09 | 0.067 | 0.133 | 0.132 |
| 29 | M | 71 | 0.116 | 0.096 | 0.095 | 0.114 |
| 30 | M | 53 | 0.087 | 0.105 | 0.074 | 0.082 |
